# Do hostile takeover threats matter? Evidence from credit ratings

**DOI:** 10.1371/journal.pone.0260688

**Published:** 2022-01-28

**Authors:** Pattanaporn Chatjuthamard, Viput Ongsakul, Pornsit Jiraporn

**Affiliations:** 1 Center of Excellence in Management Research for Corporate Governance and Behavioral Finance, SASIN School of Management, Chulalongkorn University, Bangkok, Thailand; 2 NIDA Business School, National Institute of Development Administration (NIDA), Bangkok, Thailand; 3 Securities and Exchange Commission of Thailand (SEC), Bangkok, Thailand; 4 Great Valley School of Graduate Professional Studies, The Pennsylvania State University, Malvern, Pennsylvania, United States of America; University of Brescia, ITALY

## Abstract

Exploiting a novel measure of takeover vulnerability mainly based on state legislations, we explore the effect of hostile takeover threats on credit ratings. Our results reveal that companies with more takeover exposure are assigned significantly better credit ratings. In particular, a rise in takeover vulnerability by one standard deviation results in an improvement in credit ratings by 7.89%. Our findings are consistent with the view that the disciplinary mechanism associated with the takeover market mitigates agency problems and ultimately raises firm value. Further analysis corroborates our conclusion, including propensity score matching, entropy balancing, and an instrumental-variable analysis. As our proxy for takeover susceptibility is plausibly exogenous, our results are more likely to show a causal effect.

## I. Introduction

There has been a vast literature in corporate governance. However, much of the literature focuses on internal corporate governance, such as board characteristics [1–4]. As far as external governance, the market for corporate control or the takeover market is regarded as a crucially important external governance mechanism [5–9]. We extend the literature in this area by exploring the effect of hostile takeover threats on credit ratings.

Credit ratings offer insight into a company’s default risk and financial health, thus reducing duplication of effort in financial markets. They allow investors to quickly evaluate the broad risk characteristics of a large number of firms by using a single, well-known, scale. Additionally, credit ratings are critical in regulation and private contracting as a risk-measurement and risk mitigation tool. Credit ratings are tremendously important as they have a significant influence on the cost of corporate borrowing, which is a major component of the cost of capital. Not surprisingly, there is a substantial literature on credit ratings [1, 4, 10, 11].

Exploiting a novel measure of takeover vulnerability primarily based on state legislations, we demonstrate that stronger takeover vulnerability improves credit ratings significantly. To the extent that credit ratings are a good proxy for credit risk, a potential explanation of our results is that takeover threats reduce managerial agency costs more than they exacerbate the agency costs of debt. Our results are also consistent with those reported by Cain, McKeown, and Solomon (2017) [9], who find that more takeover threats improve firm value significantly. Importantly, one crucial advantage of our study is that we rely on a novel proxy for takeover susceptibility based on plausibly exogenous factors, such as state laws [9]. Therefore, endogeneity is much less likely in our study than it is in prior research. Our results are more likely to reflect causality, rather than merely an association.

In particular, an increase in takeover susceptibility by one standard deviation results in an improvement in credit ratings by 7.89%. Consequently, the effect of takeover vulnerability on credit ratings is not only statistically significant but is also economically meaningful. We also show that stronger pressure from the takeover market results in a significantly lower probability for the firm to be assigned speculative-grade credit ratings.

While our results are already less vulnerable to endogeneity, we execute several robustness checks to further mitigate endogeneity. First, we perform propensity score matching where we match each firm in the treatment group to a firm outside the treatment group that is most comparable. Thus, our treatment and control firms are nearly identical along all observable dimensions. Our PSM results confirm that more takeover susceptibility improves credit ratings. Furthermore, we execute entropy balancing, a novel matching approach [12], which reweights each observation in the control group such that the distributions of the variables in the treatment and the control groups are similar. Third, we employ an instrumental-variable analysis using a few alternate instruments. All the robustness checks consistently corroborate our conclusion.

The results of our study contribute to the literature in several ways. First, we contribute to a vital branch of the literature that explores the takeover market as an important governance mechanism [5–9, 13, 14]. We show that credit rating agencies recognize the role of the takeover market in alleviating agency costs and assign credit ratings accordingly.

Second, our study contributes to the literature on credit ratings [1, 3, 10, 11]. While much of the literature places more emphasis on the impact of internal governance, the effect of external governance mechanisms, such as the takeover market, is much less understood. We extend the literature in this important area by showing that takeover vulnerability is one of the significant determinants of credit ratings. Moreover, our study contributes to an emerging, albeit rapidly growing, branch of the literature that exploits the hostile takeover index as an exogenous measure of takeover vulnerability [9, 13, 14].

Finally, our results contribute to an important branch of the literature that explores the agency cost of debt [15–20]. We show that the pressure associated with the takeover market does not appear to worsen the agency cost of debt. Or, at least, it does not exacerbate the agency cost of debt enough to overcome the benefits deriving from the reduction in the agency costs between shareholders and managers.

## II. Prior research and hypothesis development

### a. Credit ratings and corporate governance

Credit rating agencies regularly assess a firm’s creditworthiness based on its corporate governance principles and practices. Poor corporate governance can jeopardize a company’s ability to repay debt and may also increase the likelihood of potential losses for creditors. Most of the literature in this area concentrates on the effects of internal governance mechanisms, such as the board of directors [1–4]. However, the effects of external governance mechanisms on credit ratings are much less understood. To fill this void in the literature, we explore how credit rating agencies view the disciplinary mechanism brought about by the market for corporate control. Credit ratings are enormously important as they determine the cost of borrowing for companies. Firms with poor credit ratings experience a much higher cost of debt than those with more favorable credit ratings. For the cost of debt is a crucial component of the cost of capital, which directly affects firm performance, the importance of credit ratings could not be overemphasized. It is not surprising that there is vast literature on credit ratings [1, 3, 10, 11, 21–26].

### b. The market for corporate control and corporate governance

The takeover market or the market for corporate control is widely regarded as a critical external corporate governance mechanism [5–9]. In prior research, variations in specific takeover protections or anti-takeover laws have been employed to proxy for changes in takeover vulnerability [27–30]. Nevertheless, a significant shortcoming of previous research in this field is that each study has focused only on one or a few anti-takeover laws [9, 13, 14].

Prior research has examined the effect of the takeover market on internal governance. According to Cheng, Nagar, and Rajan (2005) [31], the adoption of second-generation anti-takeover laws results in decreased managerial ownership, meaning that the takeover market serves as a substitute for the governance role of managerial ownership in aligning managers’ and shareholders’ interests. Similarly, according to Garvey and Hanka (1999) [32], firms protected from takeover threats by second-generation anti-takeover laws exhibit less leverage, implying that the market for corporate control substitutes for the governance role of leverage [9].

Other research develops indices of takeover susceptibility based on state laws and takeover provisions at the firm level, such as the Governance Index and the Entrenchment Index [33–40]. However, these firm-level measures are highly susceptible to endogeneity issues [13, 14, 41, 42].

To resolve the issues raised in the literature, Cain et al. (2017) [9] create a hostile takeover index based on 17 takeover laws enacted between 1965 and 2014 and on plausibly exogenous variables. They demonstrate that greater takeover protection is associated with a significant decrease in firm value using this novel takeover susceptibility index, corroborating the arguments based on managerial entrenchment and agency costs. Their research is especially useful because it represents a major step forward in coping with endogeneity while also taking into consideration the full spectrum of state laws. Their findings are more likely to demonstrate causality than those in prior research [13, 14].

### c. The agency cost of debt (agency conflict between shareholders and creditors)

Although the agency conflict between managers and shareholders has attracted considerably more attention in the literature, the agency conflict between shareholders and debtholders are also crucially important, especially to credit ratings agencies. Typically, the agency cost of debt is described in terms of the risk-shifting and underinvestment problems. First, a possible conflict between equity and debt claimants occurs when shareholders expropriate debtholders’ wealth by making risky investments. As a result, debtholders do not earn a rate of return commensurate with the level of risk the are exposed to. In this case, shareholders reap most of the benefits (i.e. when high-risk projects succeed), while debtholders shoulder most of the costs [43]. This problem is also known as the asset substitution problem, where more risky assets are substituted for safer assets.

Second, managers working on behalf of shareholders can pass up positive-NPV projects if they believe that most of the benefits would accrue to debtholders. This is classically referred to as the underinvestment problem in the literature and represents suboptimal investment decisions for the company. Both risk-shifting and underinvestment give rise to the agency cost of debt [3, 4]. There is an immense literature on the agency cost of debt, highlighting the importance of this branch of the literature [15–20, 44–46].

### d. The agency cost reduction hypothesis

According to this view, weak corporate governance enables managers to become entrenched. Managerial entrenchment exacerbates agency costs by encouraging managers to take actions that favor themselves personally but do not necessarily maximize shareholder wealth. It is well established in the literature that opportunistic managers can expropriate shareholders’ wealth by a variety of means (such as excessive compensation and perquisite consumption, unnecessary acquisitions, etc.), reducing the firm’s expected cash flows and, ultimately, hurting its value [4].

However, the market for corporate control constitutes a crucial external governance mechanism that imposes discipline on managers. When subject to hostile takeover threats, managers are less inclined to take opportunistic actions that reduce shareholders’ wealth. Corroborating this argument, Cain et al. (2017) [9] report that a higher degree of takeover vulnerability raises firm value, suggesting that hostile takeover threats represent a disciplinary mechanism that alleviates the agency conflict. Because the takeover market mitigates the agency conflict and thus enhances firm value, which ultimately benefits creditors as well, credit rating agencies assign more favorable credit ratings to those companies more vulnerable to takeover threats. In conclusion, this view predicts that stronger takeover susceptibility results in better credit ratings.

### e. The risk-shifting and underinvestment hypothesis

When entrenched, managers may prefer to live a “quiet life”, avoiding difficult decisions and complex investments that demand substantial managerial efforts and time. The disciplinary mechanism triggered by an active takeover market mitigates managerial entrenchment and forces managers to engage in more risk-taking [28]. An increase in risk-taking may exacerbate the risk-shifting problem, exposing the company’s creditors to the level of risk not commensurate with their expected rate of return. Moreover, a more active takeover market worsens managerial myopia because managers are less certain about their careers when subject to more takeover threats. Consequently, they may not adopt certain positive-NPV projects that do not produce results quickly in the short run, thus exacerbating the underinvestment problem.

Furthermore, managerial entrenchment enables managers to remain in their positions for an extended period of time. This sustained presence has consequences for third parties. Longer tenure means that external parties, such as creditors, are more likely to deal with the same managers and corporate policies for an extended period of time. An exploitative behavior on the part of the managers almost certainly leads creditors to anticipate such behavior in the future as long as the same management team remains in place [4, 16]. Due to the reputation concerns, entrenched managers are less likely to act against creditors by engaging either in risk-shifting or underinvestment. This reputation mechanism, however, is significantly weakened with a more active takeover market for hostile takeover threats reduce the managers’ job security considerably. Therefore, stronger takeover susceptibility may worsen the asset substitution and underinvestment problems. In conclusion, this hypothesis argues that more takeover threats lead to poorer credit ratings.

## III. Sample formation and data description

### a. Sample formation

The data on the hostile takeover index are from Cain, McKeown, and Solomon (2017) [9]. The data on directors and board characteristics are from the Institutional Shareholder Services (ISS). The data on credit ratings and firm-specific characteristics are from COMPUSTAT. Outliers are removed where appropriate. The final sample consists of 8,533 firm-year observations from 1996 to 2014.

### b. Credit ratings

Following Klock, Mansi, and Maxwell (2005) [47], we compute credit ratings using a conversion method, where AAA-rated bonds are assigned a value of 22 and D-rated bonds are assigned a value of 1. A company with an A+ rating from S&P, for example, would earn a score of 18. We concentrate on the S&P credit ratings since several prior studies demonstrate that the S&P ratings represent the firm’s overall creditworthiness [4, 12, 48, 49].

### c. The hostile takeover index (Cain, McKeown, and Solomon, 2017)

Following Cain et al. (2017) [9], we use the hostile takeover index as a proxy for hostile takeover susceptibility. The fact that this index is focused on variables that are plausibly exogenous is a major benefit. Cain et al. (2017) [9] develop the takeover index by combining three factors: 1) legal determinants (17 state takeover laws), 2) macroeconomic factor (capital liquidity), and 3) a company-specific factor unaffected by firm choice (firm age). A higher index value indicates that the company is more vulnerable to a hostile takeover threat. As a result, this index is substantially less vulnerable to endogeneity than what has been adopted in prior research. Cain et al. (2017) [9] provide more comprehensive details about the index’s construction. This index is well adopted in the recent literature [13, 14, 50–53].

### d. Other variables

It is important to control for other factors that may affect credit ratings. Based on prior research, several control variables are included. [3, 4, 48, 49]. Specifically, we include board attributes such as board size, and the percentage of independent directors. We also include the following firm characteristics; firm size (Ln of total assets), profitability (EBIT/total assets), leverage (total debt/total assets), capital investments (capital expenditures/total assets), intangible assets (R&D/total assets and advertising expense/total assets), cash holdings (cash holdings/total assets), discretionary spending (SG&A expense/total assets, dividend payouts (total dividends/total assets), and asset tangibility (fixed assets/total assets).

Furthermore, in certain model specifications, we include firm fixed effects to control for any time-invariant firm characteristics that may be omitted from the model. We also include year and industry fixed effects to account for any variation over time and across industries (based on the first two digits of SIC). Table 1 shows the descriptive statistics for credit ratings, the hostile takeover index, and for various firm characteristics. The average credit ratings are 13.878, which approximately corresponds to BBB.

**Table 1:** Summary statistics. The hostile takeover index is from Cain, McKeown, and Solomon (2017) [9]. Credit ratings are from COMPUSTAT. Following Klock, Mansi, and Maxwell (2005), we compute credit ratings using a conversion method, where AAA-rated bonds are assigned a value of 22 and D-rated bonds are assigned a value of 1. Board size is the number of directors on the board. SG&A is selling, administrative, and general expense.

Variable	Mean	Std. Dev.	Median	25th	75th
Credit Ratings					
Credit Ratings	13.878	5.113	12.000	10.000	18.000
Takeover Susceptibility					
Hostile Takeover Index	0.206	0.105	0.190	0.119	0.288
Board Attributes					
% Independent Directors	73.579	16.114	77.778	64.286	87.500
Board Size	9.957	2.329	10.000	8.000	11.000
Firm Characteristics					
Total Assets	13142.520	39073.550	3834.198	1726.768	10051.220
EBIT/Total Assets	0.101	0.091	0.098	0.062	0.140
Total Debt/Total Assets	0.293	0.165	0.275	0.184	0.378
Capital Expenditures/Total Assets	0.057	0.058	0.039	0.023	0.069
Advertising Expense/Total Assets	0.013	0.032	0.000	0.000	0.010
R&D Expense/Total Assets	0.021	0.038	0.002	0.000	0.025
Cash Holdings/Total Assets	0.098	0.114	0.057	0.020	0.133
Dividends/Total Assets	0.015	0.023	0.009	0.000	0.021
Fixed Assets/Total Assets	0.582	0.429	0.490	0.269	0.826
SG&A Expense/Total Assets	0.198	0.175	0.154	0.071	0.277

## IV. Results

### a. Baseline regression analysis

The regression results are shown in Table 2 where the dependent variable is credit ratings. We employ an ordered logistic regression analysis as our primary estimation method with a few exceptions where it is challenging or impossible to do so. Model 1 is an ordered logistic regression with industry and year fixed effects with the standard errors clustered at the firm level. The coefficient of the hostile takeover index is positive and significant. We run an ordered logistic firm-fixed-effects regression in Model 2 to control for any time-invariant unobserved firm-specific characteristics. The coefficient of the hostile takeover index remains significantly positive. As a robustness check, we run a random-effects regression in Model 3. It is econometrically challenging to run a random-effects ordered logistic regression. So, as an approximation, we perform a random-effects regression assuming that credit ratings are a continuous variable in Model 3. The result remains consistent, suggesting that our result is robust to the estimation method.

**Table 2:** The effect of hostile takeover threats on credit ratings. The hostile takeover index is from Cain, McKeown, and Solomon (2017) [9]. Credit ratings are from COMPUSTAT. Following Klock, Mansi, and Maxwell (2005), we compute credit ratings using a conversion method, where AAA-rated bonds are assigned a value of 22 and D-rated bonds are assigned a value of 1. Board size is the number of directors on the board. SG&A is selling, administrative, and general expense.

	(1)	(2)	(3)
	Ordered Logit	Ordered Logit Firm-Fixed-Effects	Random-Effects
	Credit Ratings	Credit Ratings	Credit Ratings
**Hostile Takeover Index**	**1.611** ***	**6.937** ***	**6.509** ***
	**(2.714)**	**(2.806)**	**(7.739)**
% Independent Directors	-0.003	-0.001	-0.002
	(-0.979)	(-0.247)	(-0.462)
Ln (Board Size)	0.017	0.622	0.770***
	(0.074)	(1.332)	(2.626)
Ln (Total Assets)	0.245***	0.526**	0.761***
	(4.938)	(2.507)	(9.275)
EBIT/Total Assets	0.309	0.838	1.051**
	(0.770)	(1.083)	(1.982)
Total Debt/Total Assets	0.290	0.004	-0.602*
	(1.017)	(0.007)	(-1.670)
Capital Expenditures/Total Assets	1.797**	2.744	3.336***
	(2.220)	(1.605)	(2.791)
Advertising Expense/Total Assets	1.781	3.659	3.544
	(0.711)	(0.744)	(1.333)
R&D Expense/Total Assets	3.051**	-2.976	2.412
	(2.016)	(-0.689)	(1.104)
Cash Holdings/Total Assets	0.766**	0.215	0.783
	(2.014)	(0.269)	(1.426)
Dividends/Total Assets	13.104***	9.533**	17.304***
	(3.793)	(1.991)	(7.339)
Fixed Assets/Total Assets	0.213	1.107**	0.991***
	(1.430)	(2.156)	(4.711)
SG&A Expense/Total Assets	0.313	1.371	1.994***
	(0.591)	(1.084)	(3.276)
Constant			10.421***
			(4.047)
Industry Fixed Effects	Yes	No	Yes
Year Fixed Effects	Yes	Yes	Yes
Firm Fixed Effects	No	Yes	No
Observations	8,533	8,533	8,533
Adjusted R-squared	0.031	0.639	0.221

Robust t-statistics in parentheses.

*** p<0.01,

** p<0.05,

* p<0.1.

All the results are in favor of the agency cost reduction hypothesis, where the disciplinary mechanism imposed by the takeover market mitigates the agency conflict, resulting in less expropriation and thus higher firm value. Both shareholders and creditors are better off. As a result, companies more susceptible to takeover threats are assigned better credit ratings. Our results aptly corroborate those in Cain et al. (2017) [9], which demonstrate that stronger takeover vulnerability improves firm value. Our results refute the risk-shifting and underinvestment hypothesis, where more takeover threats worsen the agency cost of debt, leading to poorer credit ratings.

Econometrically, it is very complicated to estimate the marginal effect of a variable in an ordered logistic regression. As a close approximation, we run a firm-fixed-effects regression treating credit ratings as a continuous variable and estimate the marginal effect based on the firm-fixed-effects regression. The fixed-effects result is shown in S1 Table. We estimate the magnitude of the effect of takeover vulnerability on credit ratings as follows. One standard deviation of the hostile takeover index is 0.105. The coefficient of the hostile takeover index in the fixed-effects regression is 9.012. Therefore, a rise in takeover susceptibility by one standard deviation raises credit ratings by 0.105×9.012 = 0.946. Because credit ratings have a median of 12, an improvement of 0.946 represents 7.89% of the median. Thus, the effect of takeover susceptibility on credit ratings is not only statistically significant but is also economically meaningful.

### b. Propensity score matching (PSM)

We employ propensity score matching (PSM) to further minimize endogeneity [54, 55]. Our treatment group is comprised of companies whose hostile takeover index value is in the top quartile (indicating greatest takeover vulnerability). We identify a firm outside the treatment group that is most comparable to each firm in the treatment group based on twelve firm and board characteristics (i.e., the twelve control variables in the regression analysis). As a result, our treatment and control firms should exhibit a high degree of similarity in all measurable dimensions.

We run diagnostic testing to ensure that our matching is appropriate. Table 3 Panel A displays the results. Model 1 is a logistic regression with a binary dependent variable equal to one if the firm is in the treatment group and zero otherwise. Model 1 contains the whole sample (pre-match). The results show that the treatment firms vary greatly from the rest of the sample in many ways. In particular, the treatment firms have more independent directors, have larger board size, are larger in size, make less capital investments, invest less in R&D, hold less cash, and pay larger dividends. These material differences may complicate our analysis.

**Table 3:** Propensity score matching. The hostile takeover index is from Cain, McKeown, and Solomon (2017) [9]. Credit ratings are from COMPUSTAT. Following Klock, Mansi, and Maxwell (2005), we compute credit ratings using a conversion method, where AAA-rated bonds are assigned a value of 22 and D-rated bonds are assigned a value of 1. Board size is the number of directors on the board. SG&A is selling, administrative, and general expense.

**Panel A: Diagnostic testing**		
	(1)	(2)
	Pre-Match	Post-Match
	Treatment	Treatment
% Independent Directors	0.036***	-0.002
	(6.253)	(-0.367)
Ln (Board Size)	1.056**	0.009
	(2.463)	(0.019)
Ln (Total Assets)	0.443***	-0.015
	(4.690)	(-0.147)
EBIT/Total Assets	-0.369	-0.405
	(-0.538)	(-0.411)
Total Debt/Total Assets	-0.823	0.279
	(-1.508)	(0.399)
Capital Expenditures/Total Assets	-5.203***	-0.935
	(-2.808)	(-0.404)
Advertising Expense/Total Assets	2.288	2.268
	(0.829)	(0.646)
R&D Expense/Total Assets	-5.203*	0.196
	(-1.782)	(0.049)
Cash Holdings/Total Assets	-1.588**	0.471
	(-2.121)	(0.491)
Dividends/Total Assets	14.041***	2.925
	(3.444)	(0.838)
Fixed Assets/Total Assets	0.168	-0.054
	(0.577)	(-0.152)
SG&A Expense/Total Assets	-0.046	-0.800
	(-0.063)	(-0.988)
Constant	-9.836***	0.650
	(-5.643)	(0.313)
Industry Fixed Effects	Yes	Yes
Year Fixed Effects	Yes	Yes
Observations	8,256	4,248
**Panel B: Regression analysis**		
	(1)	(2)
	Ordered Logit	Firm-Fixed-Effects
	Credit Ratings	Credit Ratings
**Hostile Takeover Index**	**2.320** ***	**11.626** ***
	**(3.366)**	**(7.344)**
% Independent Directors	0.002	-0.006
	(0.346)	(-0.820)
Ln (Board Size)	0.205	-0.045
	(0.516)	(-0.087)
Ln (Total Assets)	0.363***	1.189***
	(5.204)	(5.814)
EBIT/Total Assets	2.452***	0.870
	(3.557)	(0.946)
Total Debt/Total Assets	-0.234	-1.213
	(-0.403)	(-1.532)
Capital Expenditures/Total Assets	2.982	12.960***
	(1.571)	(4.587)
Advertising Expense/Total Assets	-2.293	-9.268*
	(-0.742)	(-1.864)
R&D Expense/Total Assets	-0.925	-9.619*
	(-0.285)	(-1.890)
Cash Holdings/Total Assets	0.351	1.851
	(0.438)	(1.636)
Dividends/Total Assets	7.452***	24.078***
	(2.824)	(6.061)
Fixed Assets/Total Assets	0.148	1.899***
	(0.506)	(3.678)
SG&A Expense/Total Assets	1.189	3.496**
	(1.218)	(2.561)
Constant		0.656
		(0.302)
Industry Fixed Effects	No	No
Year Fixed Effects	Yes	Yes
Firm Fixed Effects	No	Yes
Observations	4,248	4,248
R-squared	0.048	0.737

Robust z-statistics in parentheses

*** p<0.01,

** p<0.05,

* p<0.1

Model 2 is a logistic regression for the propensity-score matched sample (post-match). In Model 2, none of the coefficients are statistically significant. As a result, our treatment and control firms are almost identical, statistically indistinguishable in all measurable dimensions. To the extent that takeover threats are irrelevant, the treatment and the control companies should demonstrate similar credit ratings. In Panel B of Table 3, the regression results for the propensity-score matched sample are presented. Model 1 is an ordered logistic regression analysis whereas Model 2 is a firm-fixed-effects analysis. We treat credit ratings as a continuous variable in Model 2 as it is challenging to run a firm-fixed-effects ordered logistic regression in this context with propensity score matching. The coefficients of the hostile takeover index are positive and significant in both models, reinforcing the agency cost reduction hypothesis once again.

Takeover threats keep opportunistic managers in line, preventing them from taking actions that diminish firm value. Agency costs are ameliorated as a result, making both shareholders and creditors better off. As our PSM results are consistent, our conclusion is unlikely driven by endogeneity.

### c. Entropy balancing

Earlier research relies on the restrictive assumption of selection on observables. To sidestep this assumption, we use Hainmueller’s (2012) [12] entropy balancing technique, a generalization of conventional matching approaches. Entropy balancing corrects for self-selection caused by observable characteristics by controlling for a large number of variables that may affect the treatment and control groups differently. Entropy balancing, in particular, enables a high degree of covariate balance by employing a reweighting scheme that incorporates covariate balance directly into a weight function that is applied to the sample units [12]. In practice, entropy balancing imposes a variety of balance restrictions, which means that the matching covariate distributions of the treatment and control groups in the preprocessed data match precisely on all prespecified moments [12, 56].

Entropy balancing is carried out as follows. We identify companies whose hostile takeover index is in the top quartile as our treatment group. The remainder of the sample is regarded as the control group. Then, on all of the control variables, we conduct entropy balancing to ensure that the mean, variance, and skewness of the observations in the two groups are identical. Table 4 shows the regression results based on the entropy balanced sample. The hostile takeover index carries significantly positive coefficients in both models, lending credence to the agency cost reduction hypothesis once again. In Model 2, we treat credit ratings as a continuous variable since running a firm-fixed-effects ordered logistic regression with entropy balancing is difficult in this case.

**Table 4:** Entropy balancing. The hostile takeover index is from Cain, McKeown, and Solomon (2017) [9]. Credit ratings are from COMPUSTAT. Following Klock, Mansi, and Maxwell (2005), we compute credit ratings using a conversion method, where AAA-rated bonds are assigned a value of 22 and D-rated bonds are assigned a value of 1. Board size is the number of directors on the board. SG&A is selling, administrative, and general expense.

	(1)	(2)
	Ordered Logit	Firm-Fixed-Effects
	Credit Ratings	Credit Ratings
**Hostile Takeover Index**	2.033***	8.742***
	**(3.294)**	**(7.714)**
% Independent Directors	-0.001	-0.004
	(-0.165)	(-0.899)
Ln (Board Size)	0.279	0.710**
	(0.871)	(2.015)
Ln (Total Assets)	0.336***	1.101***
	(5.615)	(7.954)
EBIT/Total Assets	2.827***	2.539***
	(4.285)	(3.245)
Total Debt/Total Assets	-0.113	-1.534***
	(-0.232)	(-2.988)
Capital Expenditures/Total Assets	3.145**	7.744***
	(2.179)	(4.323)
Advertising Expense/Total Assets	-0.782	-4.152
	(-0.287)	(-1.164)
R&D Expense/Total Assets	1.227	-8.429***
	(0.525)	(-2.580)
Cash Holdings/Total Assets	0.316	-0.482
	(0.516)	(-0.615)
Dividends/Total Assets	4.025**	6.344***
	(2.306)	(3.574)
Fixed Assets/Total Assets	0.350	1.864***
	(1.434)	(5.659)
SG&A Expense/Total Assets	1.096	4.806***
	(1.580)	(5.349)
Constant		0.851
		(0.565)
Industry Fixed Effects	No	No
Year Fixed Effects	Yes	Yes
Firm Fixed Effects	No	Yes
Observations	8,533	8,533

### d. Instrumental-variable analysis (IV)

We perform an instrumental-variable (IV) analysis to further reduce endogeneity. This method mitigates the endogeneity biases caused by reverse causality, unobserved heterogeneity, and measurement errors. The value of the hostile takeover index in the earliest year for each firm is our first instrumental variable. The idea is that the hostile takeover index in the earliest year could not have resulted from credit ratings in any of the subsequent years, making reverse causality unlikely.

Table 5 shows the regression results. Model 1 is the first-stage regression where the hostile takeover index is the dependent variable. The coefficient of the takeover index in the earliest year is significantly positive, as expected. Model 2 is the second-stage ordered logistic regression where the dependent variable is credit ratings. The coefficient of the hostile takeover index instrumented from the first stage is significantly positive. Thus, our IV results confirm that more takeover threats improve credit ratings significantly, consistent with the agency cost reduction hypothesis.

**Table 5:** Instrumental-variable analysis. The hostile takeover index is from Cain, McKeown, and Solomon (2017) [9]. Credit ratings are from COMPUSTAT. Following Klock, Mansi, and Maxwell (2005), we compute credit ratings using a conversion method, where AAA-rated bonds are assigned a value of 22 and D-rated bonds are assigned a value of 1. Board size is the number of directors on the board. SG&A is selling, administrative, and general expense. Our instrument is the value of the hostile takeover index in the earliest year for each firm.

	(1)	(2)
	First Stage	Second Stage
	OLS	Ordered Logit
	Hostile Takeover Index	Credit Ratings
**Hostile Takeover Index (Earliest Year)**	**0.751** ***	
	**(29.628)**	
**Hostile Takeover Index (Instrumented)**		**2.142** ***
		**(2.779)**
% Independent Directors	0.000	-0.003
	(1.117)	(-1.128)
Ln (Board Size)	0.005	-0.005
	(0.733)	(-0.022)
Ln (Total Assets)	0.002	0.233***
	(1.273)	(4.657)
EBIT/Total Assets	-0.011	0.326
	(-0.965)	(0.814)
Total Debt/Total Assets	-0.013*	0.306
	(-1.805)	(1.073)
Capital Expenditures/Total Assets	-0.054**	1.877**
	(-2.546)	(2.328)
Advertising Expense/Total Assets	0.045	1.813
	(1.156)	(0.723)
R&D Expense/Total Assets	-0.026	3.143**
	(-0.584)	(2.084)
Cash Holdings/Total Assets	0.025**	0.783**
	(2.196)	(2.058)
Dividends/Total Assets	0.106*	12.821***
	(1.883)	(3.708)
Fixed Assets/Total Assets	0.003	0.203
	(0.977)	(1.370)
SG&A Expense/Total Assets	-0.006	0.300
	(-0.325)	(0.576)
Constant	0.031	
	(1.490)	
Industry Fixed Effects	Yes	Yes
Year Fixed Effects	Yes	Yes
Observations	8,533	8,533
Adjusted R-squared	0.794	0.031

Robust t-statistics in parentheses.

*** p<0.01,

** p<0.05,

* p<0.1.

One potential criticism for this instrumental variable is that the hostile takeover index can be sticky over time, changing only slowly. As a result, the index’s value in the earliest year might not be significantly different from its value in any given year. We mitigate this problem by calculating the standard deviation of the hostile takeover index over time for each firm. A larger standard deviation indicates that the index has more variation over time for a given company. Then, we conduct an IV analysis on only those firms with a standard deviation greater than the median. In essence, we concentrate on companies whose takeover index changes more rapidly over time. This approach alleviates the concern about the takeover index’s stickiness.

The regression result is shown in Table 6. The coefficient of the hostile takeover index instrumented from the first stage is both positive and significant. For further robustness, we apply propensity score matching (PSM) and entropy balancing on top of the IV analysis. The results are shown in Table 7. All the results in Table 7 are consistent, supporting the prediction of the agency cost reduction hypothesis where more takeover threats improve credit ratings.

**Table 6:** Instrumental-variable analysis on the high-variance subsample. The hostile takeover index is from Cain, McKeown, and Solomon (2017) [9]. Credit ratings are from COMPUSTAT. Following Klock, Mansi, and Maxwell (2005), we compute credit ratings using a conversion method, where AAA-rated bonds are assigned a value of 22 and D-rated bonds are assigned a value of 1. Board size is the number of directors on the board. SG&A is selling, administrative, and general expense.

	(1)
	Ordered Logistic
	Second Stage
	Credit Ratings
**Hostile Takeover Index (Instrumented)**	**2.661** ***
	**(2.627)**
% Independent Directors	-0.006
	(-1.261)
Ln (Board Size)	0.265
	(0.766)
Ln (Total Assets)	0.234***
	(3.375)
EBIT/Total Assets	0.754
	(1.040)
Total Debt/Total Assets	0.426
	(1.001)
Capital Expenditures/Total Assets	2.986**
	(2.272)
Advertising Expense/Total Assets	2.825
	(0.764)
R&D Expense/Total Assets	3.606
	(1.585)
Cash Holdings/Total Assets	1.462**
	(2.404)
Dividends/Total Assets	16.047***
	(3.572)
Fixed Assets/Total Assets	0.220
	(0.804)
SG&A Expense/Total Assets	-0.183
	(-0.249)
Industry Fixed Effects	Yes
Year Fixed Effects	Yes
Observations	4,319
Adjusted R-squared	0.041

z-statistics in parentheses.

*** p<0.01,

** p<0.05,

* p<0.1.

**Table 7:** Second-stage instrumental-variable analysis with propensity score matching and entropy balancing. The hostile takeover index is from Cain, McKeown, and Solomon (2017) [9]. Credit ratings are from COMPUSTAT. Following Klock, Mansi, and Maxwell (2005), we compute credit ratings using a conversion method, where AAA-rated bonds are assigned a value of 22 and D-rated bonds are assigned a value of 1. Board size is the number of directors on the board. SG&A is selling, administrative, and general expense.

	(1)	(2)
	Propensity Score Matching	Entropy Balancing
	Ordered Logit	Ordered Logit
	Credit Ratings	Credit Ratings
**Hostile Takeover Index (Instrumented)**	**2.655** ***	**2.416** ***
	**(2.813)**	**(2.948)**
% Independent Directors	0.001	-0.001
	(0.155)	(-0.268)
Ln (Board Size)	0.160	0.242
	(0.404)	(0.754)
Ln (Total Assets)	0.348***	0.322***
	(4.886)	(5.248)
EBIT/Total Assets	2.459***	2.830***
	(3.578)	(4.261)
Total Debt/Total Assets	-0.234	-0.104
	(-0.403)	(-0.213)
Capital Expenditures/Total Assets	3.065	3.188**
	(1.624)	(2.235)
Advertising Expense/Total Assets	-2.153	-0.745
	(-0.704)	(-0.273)
R&D Expense/Total Assets	-0.824	1.250
	(-0.256)	(0.534)
Cash Holdings/Total Assets	0.414	0.374
	(0.515)	(0.604)
Dividends/Total Assets	7.225***	3.817**
	(2.642)	(2.160)
Fixed Assets/Total Assets	0.142	0.339
	(0.480)	(1.387)
SG&A Expense/Total Assets	1.101	1.068
	(1.163)	(1.559)
Year Fixed Effects	Yes	Yes
Industry Effects	Yes	Yes
Observations	4,248	8,533
Adjusted R-squared	0.048	-0.923

z-statistics in parentheses.

*** p<0.01,

** p<0.05,

* p<0.1.

To further ensure that our results are robust, we employ an alternate instrumental variable. We adopt a historical geographic instrument, which is the average hostile takeover index of all firms located in the same city in the earliest year. The idea is that firms located close by tend to be exposed to similar economic conditions. Besides, as a firm’s headquarters location is usually determined early in its life and very rarely changes [57, 58], an instrument based on geography is considered plausibly exogenous. Also, because there are many firms in a city, any variation in takeover vulnerability at the city level is plausibly exogenous to firm-specific characteristics. By using this method, our instrument is from outside the firm (average of many firms in the same city) and from outside the current time period (the earliest year, rather than the current year), making it more likely exogenous. This technique of using a geographic instrument has been adopted in recent research [49, 58–60].

The regression results are shown in Table 8. Model 1 is the first-stage regression where the dependent variable is the hostile takeover index. The coefficient of the average takeover index of all firms located in the same city in the earliest year is significantly positive as expected. Model 2 is the second-stage regression where the dependent variable is credit ratings. The coefficient of the hostile takeover index instrumented from the first stage is positive and significant. All the results in Table 8 reinforce the agency cost reduction hypothesis, showing that stronger takeover susceptibility raises credit ratings significantly. Our results appear to be quite robust.

**Table 8:** Instrumental-variable analysis using a historical geographic instrument with propensity score matching and entropy balancing. The hostile takeover index is from Cain, McKeown, and Solomon (2017) [9]. Credit ratings are from COMPUSTAT. Following Klock, Mansi, and Maxwell (2005), we compute credit ratings using a conversion method, where AAA-rated bonds are assigned a value of 22 and D-rated bonds are assigned a value of 1. Board size is the number of directors on the board. SG&A is selling, administrative, and general expense.

	(1)	(2)
	First Stage	Second Stage
	OLS	Ordered Logit
	Whole Sample	Whole Sample
	Hostile Takeover Index	Credit Ratings
**Hostile Takeover Index (Earliest City-Average)**	**0.699** ***	
	**(26.179)**	
**Hostile Takeover Index (Instrumented)**		**2.194** **
		**(2.301)**
% Independent Directors	0.000***	-0.003
	(3.379)	(-1.165)
Ln (Board Size)	0.024**	-0.017
	(2.476)	(-0.073)
Ln (Total Assets)	0.007***	0.230***
	(3.367)	(4.468)
EBIT/Total Assets	-0.015	0.314
	(-0.923)	(0.794)
Total Debt/Total Assets	-0.022**	0.314
	(-2.006)	(1.092)
Capital Expenditures/Total Assets	-0.103**	1.893**
	(-2.551)	(2.330)
Advertising Expense/Total Assets	-0.136*	1.935
	(-1.932)	(0.795)
R&D Expense/Total Assets	-0.107**	3.175**
	(-2.087)	(2.108)
Cash Holdings/Total Assets	0.026*	0.784**
	(1.753)	(2.043)
Dividends/Total Assets	0.253***	12.891***
	(2.688)	(3.670)
Fixed Assets/Total Assets	0.003	0.201
	(0.442)	(1.334)
SG&A Expense/Total Assets	0.018	0.298
	(1.167)	(0.560)
Constant	-0.142***	
	(-3.371)	
Year Fixed Effects	Yes	Yes
Industry Fixed Effects	Yes	Yes
Observations	8,533	8,533
Adjusted R-squared/Pseudo R-squared	0.604	0.031

Robust t-statistics in parentheses.

*** p<0.01,

** p<0.05,

* p<0.1.

### e. Speculative-grade credit ratings

It could be argued that there is a possible threshold effect where companies rated above speculative grades may enjoy a much lower cost of debt. Some financial institutions are not allowed to invest in debt instruments that are below a certain threshold. So, the effect around the threshold may be distinct from the effect elsewhere in the distribution. To gain further insights, we construct a binary variable labelled “Junk”, which is equal one if the firm’s credit ratings are below BBB, and zero otherwise.

Table 9 shows the regression results where the dependent variable is the binary variable, Junk. Model 1 is a logistic regression. The hostile takeover index carries a negative and significant coefficient. Model 2 is a firm-fixed-effects logistic regression, which controls for time-invariant unobservable characteristics. Again, the coefficient of the hostile takeover index is significantly negative. Finally, Model 3 is a logistic regression with propensity score matching. The coefficient of the takeover index remains negative and significant. All the results imply that stronger pressure from the takeover market lowers the probability of having speculative-grade credit ratings significantly. We calculate the marginal effect and find that a rise in takeover susceptibility by one standard deviation reduces the probability of being assigned speculative-grade ratings by 3.32%.

**Table 9:** The effect of takeover susceptibility on the speculative-grade status. The hostile takeover index is from Cain, McKeown, and Solomon (2017) [9]. Credit ratings are from COMPUSTAT. Following Klock, Mansi, and Maxwell (2005), we compute credit ratings using a conversion method, where AAA-rated bonds are assigned a value of 22 and D-rated bonds are assigned a value of 1. Board size is the number of directors on the board. SG&A is selling, administrative, and general expense. The binary variable, Junk, is equal to one if the firm’s credit ratings are below BBB, and zero otherwise.

	(1)	(2)	(3)
	Logit	Firm-Fixed-Effects	Logit
		Logit	PSM
	Junk	Junk	Junk
**Hostile Takeover Index**	**-1.076** *	**-3.510** ***	**-1.679** **
	**(-1.777)**	**(-4.222)**	**(-2.018)**
% Independent Directors	0.006*	0.001	0.008
	(1.648)	(0.423)	(1.319)
Ln (Board Size)	0.128	-0.078	0.233
	(0.482)	(-0.280)	(0.465)
Ln (Total Assets)	-0.249***	-0.135*	-0.535***
	(-4.392)	(-1.748)	(-6.052)
EBIT/Total Assets	0.894	0.768	-3.691**
	(1.582)	(1.412)	(-2.430)
Total Debt/Total Assets	-0.517	-0.580*	0.733
	(-1.497)	(-1.722)	(1.089)
Capital Expenditures/Total Assets	-1.713	-2.067*	-2.142
	(-1.620)	(-1.916)	(-0.910)
Advertising Expense/Total Assets	-2.691	-8.742***	0.635
	(-1.273)	(-3.059)	(0.219)
R&D Expense/Total Assets	-4.524***	-7.064***	-5.266
	(-2.592)	(-3.232)	(-1.376)
Cash Holdings/Total Assets	-1.142**	-0.866	-0.534
	(-2.406)	(-1.628)	(-0.547)
Dividends/Total Assets	-17.905***	-9.415***	-21.657**
	(-4.294)	(-3.738)	(-2.430)
Fixed Assets/Total Assets	-0.323*	-0.834***	-0.362
	(-1.679)	(-4.224)	(-0.999)
SG&A Expense/Total Assets	-0.750	-0.868	-1.422
	(-1.588)	(-1.602)	(-1.529)
Constant	-0.036	2.759***	4.391***
	(-0.049)	(3.333)	(2.831)
Year Fixed Effects	Yes	Yes	Yes
Industry Fixed Effects	Yes	No	Yes
Firm Fixed Effects	No	Yes	No
Observations	8,495	8,533	4,216
Pseudo R-squared	0.088		0.198

Robust z-statistics in parentheses.

*** p<0.01,

** p<0.05,

* p<0.1.

## V. Conclusions

Exploiting a novel measure of takeover susceptibility recently invented by Cain et al. (2017) [9], we investigate how credit rating agencies view hostile takeover threats. We document that credit rating agencies view hostile takeover susceptibility favorably, assigning higher credit ratings to companies more exposed to takeover threats. Our results corroborate the agency cost reduction hypothesis. To the extent that credit ratings are a reasonable proxy for credit risk, one possible explanation for our findings is that takeover threats lower managerial agency costs more than they aggravate the agency costs of debt. That is why companies with more takeover exposure enjoy better credit ratings.

Importantly, it should be noted that our metric of takeover vulnerability is principally based on state legislations, which are plausibly exogenous to firm-specific attributes. So, our results are not likely driven by endogeneity and are much more likely to show causality, rather than merely an association. In any event, we execute several robustness checks to further reduce endogeneity. In particular, we perform propensity score matching (PSM), entropy balancing, and an instrumental-variable analysis. Our results survive all the robustness tests and are therefore highly robust.

Credit ratings are crucial as they determine the cost of borrowing for companies. So, we contribute to the literature on credit ratings by showing that takeover exposure is one of the most significant determinants of credit ratings. We also extend the literature on corporate governance by demonstrating that external governance, such as the takeover market, is viewed favorably by credit rating agencies, who assign better credit ratings to those more subject to takeover threats. One possible avenue of research that researchers can pursue in the future is to investigate the effect of takeover vulnerability on credit spreads. Such research would aptly complement our findings in this study.

## Supporting information

S1 Table Firm-fixed-effects analysis of the effect of takeover threats on credit ratings. (DOCX)
