# Supplementary material for: Do hostile takeover threats matter? Evidence from credit ratings
Source: PLoS One. 2022 Jan 28;17(1):e0260688. doi: 10.1371/journal.pone.0260688 (PMC8797189; doi:10.1371/journal.pone.0260688)
Supplement: S1 Table — (DOCX) [file pone.0260688.s001.docx]

**S1 Table: Firm-fixed-effects analysis of the effect of takeover threats on credit ratings**

|  | (1) |
| --- | --- |
|  | Credit Ratings |
|  |  |
| **Hostile Takeover Index** | **9.012***** |
|  | **(7.550)** |
| % Independent Directors | -0.001 |
|  | (-0.329) |
| Ln (Board Size) | 0.892*** |
|  | (2.774) |
| Ln (Total Assets) | 0.896*** |
|  | (7.171) |
| EBIT/Total Assets | 0.978* |
|  | (1.739) |
| Total Debt/Total Assets | -0.525 |
|  | (-1.314) |
| Capital Expenditures/Total Assets | 3.217** |
|  | (2.509) |
| Advertising Expense/Total Assets | 2.098 |
|  | (0.637) |
| R&D Expense/Total Assets | -2.014 |
|  | (-0.722) |
| Cash Holdings/Total Assets | 0.380 |
|  | (0.606) |
| Dividends/Total Assets | 14.059*** |
|  | (5.711) |
| Fixed Assets/Total Assets | 1.388*** |
|  | (5.436) |
| SG&A Expense/Total Assets | 2.610*** |
|  | (3.389) |
| Constant | 2.231* |
|  | (1.796) |
| Firm Fixed Effects | Yes |
| Year Fixed Effects | Yes |
| Pseudo R-squared | 0.639 |
| Observations | 8,533 |
| t-statistics in parentheses | |
| *** p<0.01, ** p<0.05, * p<0.1 | |
